# Why does malaria transmission continue at high levels despite universal vector control? Quantifying persistent malaria transmission by *Anopheles funestus* in Western Province, Zambia

**DOI:** 10.1186/s13071-024-06457-5

**Published:** 2024-10-14

**Authors:** Ruth A. Ashton, Benjamin Chanda, Chama Chishya, Rayford Muyabe, Tresford Kaniki, Patricia Mambo, Mwansa Mwenya, Gift Mwaanga, Annie Arnzen, Erica Orange, Kochelani Saili, Handrinah Banda Yikona, John Chulu, Chanda Chitoshi, Irene Kyomuhangi, John Miller, Kafula Silumbe, Busiku Hamainza, Megan Littrell, Joshua Yukich, Immo Kleinschmidt, Javan Chanda, Joseph Wagman, Thomas P. Eisele

**Affiliations:** 1 Centre for Applied Malaria Research and Evaluation, Tulane School of Public Health and Tropical Medicine, 1440 Canal Street, New Orleans, LA 70112 USA; 2 PATH, Kaoma, Zambia; 3 Macha Research Trust, Choma, Zambia; 4 PATH, Seattle, WA USA; 5 Centre for Health Informatics Computing and Statistics, Lancaster University, Lancaster, UK; 6 PATH, Lusaka, Zambia; 7 National Malaria Elimination Centre, Lusaka, Zambia; 8 PATH, Washington DC, USA; 9 London School of Hygiene and Tropical Medicine, London, United Kingdom

## Abstract

**Background:**

Some settings continue to experience a high malaria burden despite scale-up of malaria vector control to high levels of coverage. Characterisation of persistent malaria transmission in the presence of standard control measures, also termed residual malaria transmission, to understand where and when individuals are exposed to vector biting is critical to inform refinement of prevention and control strategies.

**Methods:**

Secondary analysis was performed using data collected during a phase III cluster randomized trial of attractive targeted sugar bait stations in Western Province, Zambia. Two seasonal cohorts of children aged 1–14 years were recruited and monitored monthly during the malaria transmission season, concurrent with entomological surveillance using a combination of human landing catch (HLC) and Centres for Disease Control (CDC) light traps at randomly selected households in study clusters. Behavioural data from cohort participants were combined with measured *Anopheles funestus* landing rates and sporozoite positivity to estimate the human behaviour-adjusted entomological inoculation rate (EIR).

**Results:**

Behavioural data from 1237 children over 5456 child-visits in 20 entomology surveillance clusters were linked with hourly landing rates from 8131 female *An*. *funestus* trapped by HLC. Among all *An*. *funestus* tested by enzyme-linked immunosorbent assay (ELISA), 3.3% were sporozoite-positive. Mean EIR directly measured from HLC was 0.07 infectious bites per person per night (ib/p/n). When accounting for child locations over the evening and night, the mean behaviour-adjusted EIR was 0.02 ib/p/n. Children not sleeping under insecticide-treated nets (ITNs) experienced 13.6 infectious bites per person per 6 month season, 8% of which occurred outdoors, while ITN users received 1.3 infectious bites per person per 6 month season, 86% of which were received outdoors. Sleeping under an ITN can prevent approximately 90% of potential *An*. *funestus* bites among children.

**Conclusions:**

In this setting ITNs have a high personal protective efficacy owing to peak *An*. *funestus* biting occurring indoors while most individuals are asleep. However, despite high household possession of ITNs (>90%) and high individual use (>70%), children in this setting experience more than one infectious bite per person per 6 month transmission season, sufficient to maintain high malaria transmission and burden. New tools and strategies are required to reduce the malaria burden in such settings.

**Graphical Abstract:**

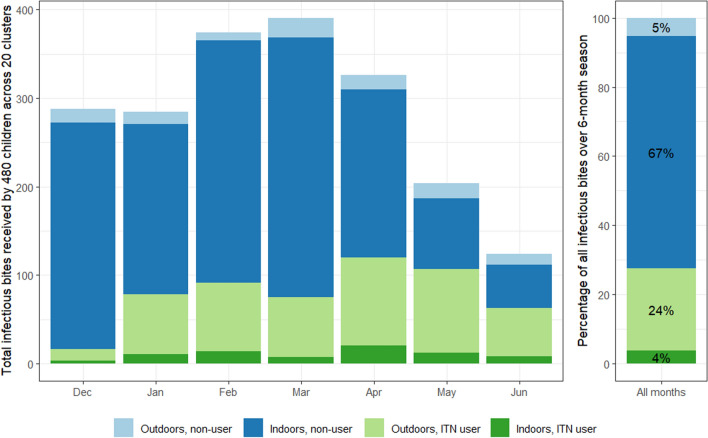

**Supplementary Information:**

The online version contains supplementary material available at 10.1186/s13071-024-06457-5.

## Background

The impressive gains made against malaria following scale-up of insecticide treated nets (ITNs) from 2000 to 2015 [1] have stagnated [2], generating concern among the malaria and wider public health community [3, 4]. It is likely that the contributing factors to this stagnation vary across geographies, but that they may include insecticide and drug resistance, changes in the dominant *Anopheles* vector species, under-resourcing of malaria control programs and broader health systems, and conflict and population displacement. There is a critical need for data from settings experiencing malaria resurgence or ongoing persistent high transmission despite adherence to current best practices in vector control and case management. Understanding the reasons for residual transmission in a given setting is likely to be critical to choosing appropriate methods and interventions to reduce it.

A recently completed phase III trial of attractive targeted sugar bait (ATSB) stations in western Zambia found only a small effect of the intervention on clinical and entomological outcomes [5, 6] in a site with high transmission despite high coverage with standard of care vector control tools. These data provide an opportunity for an in-depth examination of potential contributors to persisting high malaria burden in this population. The trial site in Western Province, Zambia is characterized by seasonal and high malaria transmission, with A*nopheles funestus* sensu stricto *(s.s)* as the primary vector*.* High vector control coverage during the trial included a mosaic approach [7] of either ITNs (a mixture of deltamethrin (Permanet 2.0) or alpha cypermethrin plus piperonyl butoxide (PBO, Veeralin) types) or indoor-residual spray (IRS, Fludora (R) Fusion, clothianidin and deltamethrin). There is a high level of pyrethroid resistance at the study site, but no evidence of resistance against clothianidin [6]. Overall, at the trial site, >70% of the population reported sleeping under an ITN, while 30% of households received IRS [5]. In total, 97% of households either owned at least one ITN or received IRS in the prior 12 months [5].

Residual malaria transmission is defined by the World Health Organization as malaria transmission which persists following the implementation in time and space of a widely effective malaria program [8]. Residual malaria transmission is not necessarily an indication that the vector population has developed physiological resistance to insecticides used in ITN and/or IRS; it can also be a result of vector behaviours which limit the potential impact of these tools or combinations of these factors [9]. These behaviours may include feeding on humans while they are outdoors and unprotected by personal protection, feeding intermittently on animals, resting outdoors, or avoiding or minimizing contact with insecticide-treated surfaces when inside households [9]. In the western Zambian context where *An*. *funestus* is anthropophilic with human blood index of 70% [10], outdoor biting and biting indoors during hours when people are not usually asleep are considered to be behaviours contributing to residual transmission. In this manuscript, we propose that ‘persistent’ malaria transmission is a more suitable term than ‘residual’ transmission, reflecting both the scale of transmission despite high coverage with existing vector control tools and the urgent need for action.

A systematic review indicates that while the proportion of bites by major malaria vectors that occur during the hours when people are usually in bed or inside are relatively high (79% and 88%, respectively), this proportion has decreased since 2000, indicating an increased proportion of biting occurring outdoors and a greater role of outdoor biting in persistent malaria transmission [11]. Urbanisation may also be related to increased time spent outside in the evenings and increased exposure to biting [12]. Sherrard-Smith et al. estimate that a 10% increase in outdoor biting could increase the entomological inoculation rate (EIR) by, on average, 0.46 infectious bites per person per year [11], although the scale of change in EIR is highly variable according to local context.

Critical to accurately estimating persistent malaria transmission is combining mosquito biting and human behaviours by time and space. EIR are often presented as direct estimates of the number of infective malaria vectors landing on a human landing collector each hour, while they sit unprotected inside or outside a home. Combining landing rates generated from human landing catch (HLC) with reported or observed human behaviour can generate estimates of biting rates that more accurately represent the bites received by community members as they follow their usual domestic routines [13, 14], which we term behaviour-adjusted EIR. Recent studies in western Kenya have reported biting by *An*. *funestus* in the early morning hours when people wake and begin daily activities [15], and late morning biting in schools [16], both potentially contributing to persistent malaria transmission. Previous estimates of EIR in Zambia not accounting for human behaviour include 4.4 indoor and 7.2 outdoor *An*. *funestus* infectious bites per person per year in south-eastern Zambia [17], while a study in northern Zambia estimated EIR of over 40 infectious *An*. *funestus* bites per person per 6 months at some study sites [18].

The objectives of this secondary analysis of entomological and human behaviour data collected during the ATSB trial were to (1) describe the proportion of *An*. *funestus* bites received by study participants that are preventable by use of ITNs, (2) estimate the behaviour-adjusted EIR in ITN-users and non-users and (3) describe the extent of persistent malaria transmission in this setting despite the high coverage of vector control.

## Methods

### Study site and design

This study is a secondary analysis of data collected as part of the phase III cluster randomized trial of Westham Sarabi ATSB stations in Western Province, Zambia [19, 20]. Briefly, the trial aimed to assess the impact of ATSB stations on clinical malaria incidence among children aged 1–14 years in the context of high standard of care vector control coverage. A total of 70 clusters were designed for the purpose of the trial in Luampa, Kaoma and Nkeyema districts in Western Province, Zambia, and assigned 1:1 to receive the intervention (two ATSB stations deployed on each eligible residential structure) or control (no ATSB). A full description of the Zambia trial site is available elsewhere [7]. The whole trial area received the standard of care vector control according to Zambia national policy: at the time of the trial this involved assignment of health facility catchments to receive either ITN or IRS [21]. In addition, two supplemental ITN distributions were conducted during the trial period to ensure high household coverage of ITNs: one Permanet 2.0 net provided to each household across the whole study site in February 2022, and one Veeralin PBO net per two household residents in September 2022 to the 48 clusters not fully covered by IRS. Across the study site, 95% of households owned at least one ITN, and 67% households had at least one ITN for every two people or IRS [5].

Data sources in the current analysis include a seasonal cohort of children aged 1–14 years, and monthly entomological surveillance by human landing catch methods, described further below. Behavioural data from the cohort dataset were combined with mean *An*. *funestus* landing rates and sporozoite positivity to estimate biting rates, described further in the analysis section.

Throughout this paper ‘directly measured’ biting rates and EIR refer to the estimates generated directly by exposed HLC collectors. ‘Human behaviour-adjusted’ biting rates and EIR refer to the estimated biting rates that would be experienced by individuals taking account of their actual location and behaviours at different times of the night.

### Entomology procedures

Entomology surveillance was conducted in 20 of the 70 trial clusters, ten in the intervention arm and ten in control arm. HLC were conducted each month at ten randomly selected households per cluster for one night with monthly replacement of households (200 collection-nights per month across all 20 clusters). Sampling and collection procedures are described in detail elsewhere [6]. Mosquito collections were conducted monthly from November 2021 to June 2022 (trial year 1), and from November 2022 to June 2023 (trial year 2). Owing to coronavirus disease 2019 (COVID-19) mitigation strategies, indoor mosquito collections were suspended in January and February 2022.

Selected households were visited to explain collection procedures and seek consent to conduct HLC collections. Between the hours of 18:00 to 06:00, one collector was seated inside the house and a second collector outside within 5–10 m of the same sleeping structure. Each collector used a flashlight to find any host seeking mosquitoes landing on their exposed lower legs and used a mouth aspirator to collect the mosquitoes and transfer to a collection cup. Collectors worked for 45 min in each hour period. Collection cups for each household, hour and indoor or outdoor location were labelled with unique codes.

Additional mosquito collection using Centres for Disease Control (CDC) light traps (CDC Miniature Downdraft Blacklight UV Light Traps, Model 912, John W. Hock Co., Gainesville FL) was conducted, with one indoor and one outdoor trap set up at a house neighbouring each HLC household. Mosquito densities from CDC light trap collections are reported elsewhere [6]. For the current analysis, morphologically identified female *An*. *funestus* sensu lato (*s*.*l*.) from CDC light trap collections were combined with *An*. *funestus* *s*.*l*. from HLC for sporozoite detection, but are not otherwise included in analysis. As detailed in Wagman et al., 93.5% of the 3522 *An*. *funestus* *s*.*l*. specimens tested by PCR were confirmed as *An*. *funestus sensu stricto* [6]. Since not all specimens included in this analysis had molecular species confirmation, we report results among *An*. *funestus* *s*.*l*.

Mosquitoes collected using HLC method were transferred to the field laboratory alive shortly after 06:00 h, knocked down by mechanical shaking of collection cups, then all *Anopheles* were morphologically identified to species or species group using the appropriate dichotomous key [22]. All *Anopheles* spp. mosquitoes were stored on silica gel in individual microcentrifuge tubes. A subsample of *Anopheles* underwent enzyme-linked immunosorbent assay (ELISA) analysis to screen for sporozoites at laboratories at the Macha Research Trust in Choma and National Malaria Elimination Center in Lusaka using standard sandwich ELISA methods to detect *P*. *falciparum* circumsporozoite protein [23].

### Cohort procedures

Two seasonal cohorts were enrolled and followed for up to 6 months, coinciding with the rainy and peak malaria transmission season. Enrolment took place in November–December 2021 and 2022, and scheduled monthly follow-up visits took place from January to June 2022 and 2023. Within each of the 70 trial clusters a target of 35 children aged 1–14 years were invited to participate in the cohort, with random selection and recruitment repeated for the second season. Full details of cohort recruitment procedures and eligibility criteria are described elsewhere [5].

At the enrolment visit and each monthly follow-up, a short questionnaire was completed to collect information on the child’s schedule and ITN use on the previous night. At the enrolment visit, additional questionnaire sections were completed to collect additional demographic and socio-economic indicators, as well as information on household and sleeping structure construction.

### Analytic approach

#### Sporozoite positivity

Sporozoite screening results were restricted to *An*. *funestus*, since *An*. *funestus* *s*.*l*. were responsible for over 95% of infectious bites at the study site [6]. Owing to small denominators, it was not possible to describe sporozoite positivity across more than one time or place classification. Consequently, two separate sporozoite summary measures were generated: (i) sporozoite positive by collection hour, generated by pooling samples from HLC collections over all clusters and both study years to generate a single study-wide estimate for each trapping hour from 18:00 to 05:00, and (ii) sporozoite positivity by cluster, generated by pooling samples from both HLC and CDC light trap collections over both study years and all collection hours to generate a single estimate for each cluster.

#### Generating directly-measured biting rates and EIR

Entomology surveillance data from HLCs were rescaled to estimate landing numbers for a full hour collection period (collectors rested for the last 15 min of each hour). To estimate mean nightly *An*. *funestus* landings, total *An*. *funestus* collected each night at each household were calculated separately for indoor and outdoor collections, then the mean of indoor and outdoor collections was generated for each household and trap night. The overall cluster mean nightly *An*. *funestus* landings was the mean of sum total landings across all households and trap nights from the specific cluster over all collection months. Directly-measured nightly EIR was estimated by multiplying the cluster mean nightly landing rate by the cluster-specific proportion sporozoite positive. To generate directly-measured EIR per person per 6 month transmission season, month-specific mean nightly landing rates were generated for January–June inclusive, multiplied by the proportion sporozoite positive, then inflated by the respective number of days in the calendar month and summed over the 6 month period.

#### Generating human behaviour-adjusted biting rates and EIR

Cohort data were restricted to the 20 clusters which participated in entomology surveillance, and to those individuals in each follow-up visit with complete information describing: the time they entered the sleeping structure at night and exited the sleeping structure in the morning, if they slept under an ITN the previous night, and if an ITN user, what time they went under the ITN at night and exited the ITN in the morning. For each child follow-up, reported ITN use and times to enter/exit the sleeping structure and ITN were used to classify the proportion of each hour between 18:00 to 06:00 that each child was (i) outside, (ii) inside and under an ITN and (iii) inside but not under an ITN. ITNs were not reported to be used outdoors in this setting. Children who spent any time sleeping under an ITN on the night before the visit were classed as ITN users for the specified calendar month. Cohort visits conducted in January and February 2022 were excluded as a result of COVID-19-related suspension of indoor HLC in those 2 months.

Entomology surveillance data from HLCs were rescaled to estimate landing numbers for a full hour collection period. Mean *An*. *funestus* landing rates were generated separately for indoor and outdoor locations for each specific hour-, month-, year-, and cluster combination. The cohort dataset describing the proportion of each hour each child spent outdoors, indoors but not under an ITN, and under an ITN was linked with the relevant mean *An*. *funestus* landing rate for the outdoor/indoor location and specific hour, cluster, month, and year. It was assumed that zero bites were experienced while under an ITN. Estimated bites accrued in each hour of the period 18:00 to 06:00 were aggregated to generate the total bites experienced by each child on each visit, and the proportion of all bites which occurred outdoors.

Human behaviour-adjusted nightly EIR within each cluster was estimated by multiplying child-specific total bites per night by cluster-specific proportion sporozoite positive, then aggregating to mean behaviour adjusted EIR for each cluster. Behaviour-adjusted bites experienced per 6 month season were estimated from the month-specific bites experienced by children within each cluster, inflated by the respective number of days in the calendar month, then summed to a total for the 6 month period from January to June.

All analyses were performed in R version 4.2.2 (R Foundation for Statistical Computing, Vienna, Austria). Infectious biting rate uncertainties were propagated from the relevant biting rate and sporozoite positivity estimates using first-order Taylor series method, using the ‘errors’ package in R [24].

### Ethical considerations

Written informed consent was collected from households where HLC took place, indoor collections were conducted in structures where sleeping residents were the same gender as the mosquito collector. Collectors were trained community members who underwent malaria testing by RDT to confirm they were free from malaria prior to each monthly collection round, and then received a course of dapsone-pyrimethamine (Deltaprim™, Zimbabwe Pharmaceuticals Ltd) to protect them from *P*. *falciparum* during HLC. Collectors testing positive by RDT received a full course of artemether lumefantrine and were excluded from that monthly collection round. Written informed consent for participation of children in the cohort was provided by a parent or guardian, and children aged 7 years and older provided written assent. Data presented in this manuscript were collected as part of the phase III ATSB trial in Zambia, which received ethical approval from the University of Zambia Biomedical research ethics committee (ref # 1197-2020), PATH REC (ref # 1460046-5) and Tulane University (ref # 2019-595). The trial is registered at ClinicalTrials.gov (NCT04800055).

## Results

### Reported evening schedule among cohort participants

Of the 4494 children participating in the cohort study across the whole trial site, 1237 were resident in the 20 entomological surveillance clusters and had complete information on all visits on structure entry/exit times and ITN use the previous night. This dataset comprised 5456 total child-visits when including enrolment visits, or 4857 visits during the transmission season from January to June.

Children under 5 years were generally inside their sleeping structures between 20:00 and 07:00 (Table 1). Older children tended to go inside slightly later in the evening and rise a little earlier in the morning. Among those children who were reported to have slept under an ITN (76.5%), over 70% report going under their ITN immediately after entering the sleeping structure. Similarly, in the morning most children did not remain in their sleeping structure after exiting their ITN, but immediately went outside. There were no reports of outdoor sleeping overnight among study children. Reported ITN use varied by month: being lowest in November–December prior to the rainy season, and highest from March to June during the peak of malaria transmission.

**Table 1:** Description of evening schedule and ITN use among children in the 20 entomology clusters

Indicator	<5 years	5–9 years	10–14 years
Number children	423	490	324
Number child-visits	2058	2391	1658
Child-visits where child reported using ITN (%)	1776 (86.3)	1854 (77.5)	1062 (64.1)
Median time went inside sleeping structure at night (IQR)	20:00 (19:00–20:00)	20:00 (19:30–20:30)	20:00 (20:00–21:00)
Median time exited sleeping structure in the morning (IQR)	07:00 (06:00–07:15)	07:00 (06:00–07:00)	06:30 (06:00–07:00)
Number (%) child-visits where child was inside sleeping structure at or before:			
18:00	121 (5.9)	45 (1.9)	28 (1.7)
19:00	707 (34.4)	478 (20.0)	223 (13.4)
20:00	1659 (80.6)	1662 (69.5)	913 (55.1)
21:00	1998 (97.1)	2252 (94.2)	1438 (86.7)
22:00	2050 (99.6)	2379 (99.5)	1622 (97.8)
% Child-visits where child was outside sleeping structure at or before:			
05:00	29 (1.4)	16 (0.7)	43 (2.6)
06:00	570 (27.7)	636 (26.6)	696 (42.0)
07:00	1469 (71.4)	1817 (76.0)	1378 (83.1)
08:00	1955 (95.0)	2314 (96.8)	1604 (96.7)
Among ITN users, number reported going under ITN immediately after entering sleeping structure (%)	1290 (72.6)	1399 (75.5)	772 (72.7)
Among ITN users not going under ITN immediately after entering sleeping structure, median (IQR) time spent indoors before going under ITN	30 min (10–60)	30 min (10–60)	30 min (15–60)
Among ITN users, number reported exiting structure immediately after leaving ITN (%)	1327 (74.7)	1445 (77.9)	792 (74.6)
Among ITN users remaining in sleeping structure after exiting ITN, median (IQR) time spent indoors after leaving ITN	25 min (8–60)	20 min (7–43)	27 min (10–45)
Number (%) reporting ITN use previous night by visit round:			
Nov/Dec	140 (71.8)	138 (58.2)	73 (43.7)
January	189 (78.1)	198 (68.5)	105 (51.2)
February	231 (78.6)	243 (69.0)	125 (49.8)
March	296 (89.2)	290 (78.2)	179 (68.6)
April	299 (92.3)	332 (86.5)	192 (76.5)
May	310 (92.5)	331 (86.6)	197 (76.4)
June	311 (92.6)	322 (85.6)	191 (72.1)
Consistency of ITN use over all visit rounds:			
Consistent ITN user	271 (64.0)	248 (50.6)	104 (32.1)
Inconsistent ITN user	146 (34.0)	205 (41.8)	182 (56.2)
Never used ITN	6 (1.4)	37 (7.5)	38 (11.7)

### Summary of *An*. *funestus* landing rates and directly-measured EIR across the study site

A total of 8233 *An*. *funestus* (8131 female and 102 male) were trapped by HLC over a total of 2968 location-nights. The following results describe female *An*. *funestus* only.

**Fig. 1:**
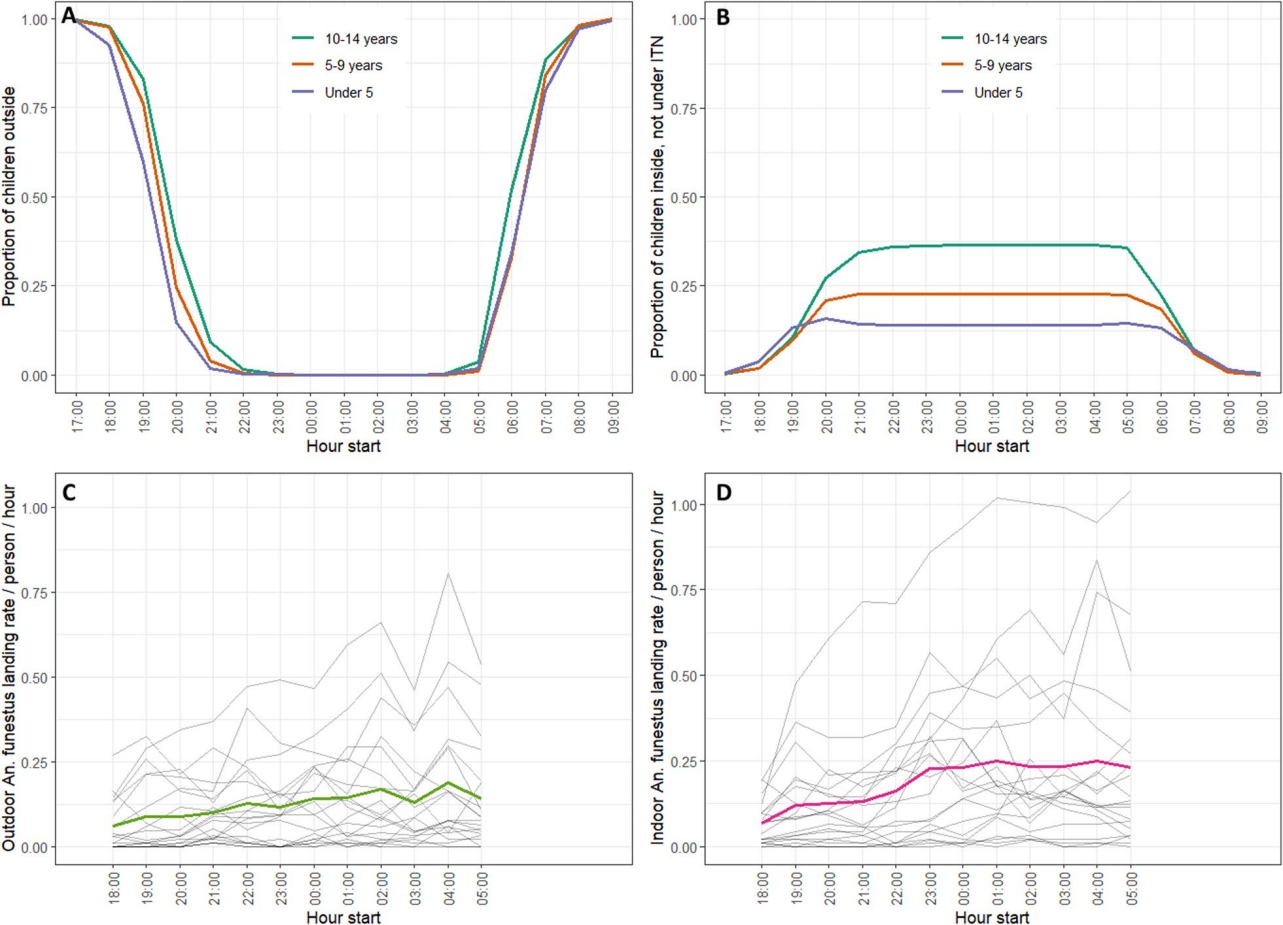
Summary of child location by hour and mean *An*. *funestus* landing on human landing collectors by hour. **A** Proportion of children remaining outside by hour and age group. **B** Proportion of children inside their sleeping structure but not under ITN by hour and age group. **C** Hourly outdoor *An*. *funestus* landing rates per HLC collector by cluster (grey lines) and mean hourly *An*. *funestus* outdoor landing rate per person across all clusters (green line). **D** Hourly indoor *An*. *funestus* landing rates per HLC collector by cluster (grey lines) and mean hourly outdoor landing rate per person across all clusters (pink line)

When pooling data from all 20 clusters, the indoor *An*. *funestus* landing rate was highest at 04:00–05:00 with a mean 0.25 *An*. *funestus* bites per person per hour (b/p/h). Landing rates were relatively consistent indoors, with 0.23–0.25 b/p/h between 23:00 and 06:00 (Fig. 1). Mean outdoor landing rates were lower than indoor landing rates, peaking at 04:00–05:00 with 0.19 b/p/h. Outdoor hourly landing rates show a gradual increase from 18:00 to midnight, then are relatively consistent between midnight and 06:00.

Both indoor and outdoor hourly landing rates showed wide variation by cluster. The highest recorded indoor and outdoor landing rates were observed in the same cluster at similar times: peak indoor biting was 1.04 b/p/h from 05:00 to 06:00 and peak outdoor biting was 0.81 b/p/h between 04:00 and 05:00 (Fig. 1). Mean nightly *An*. *funestus* landing rate was also highly variable across clusters, from 0.10 to 7.45 bites per night experienced by HLC collectors while exposed to bites during the period 18:00 to 06:00.

**Fig. 2:**
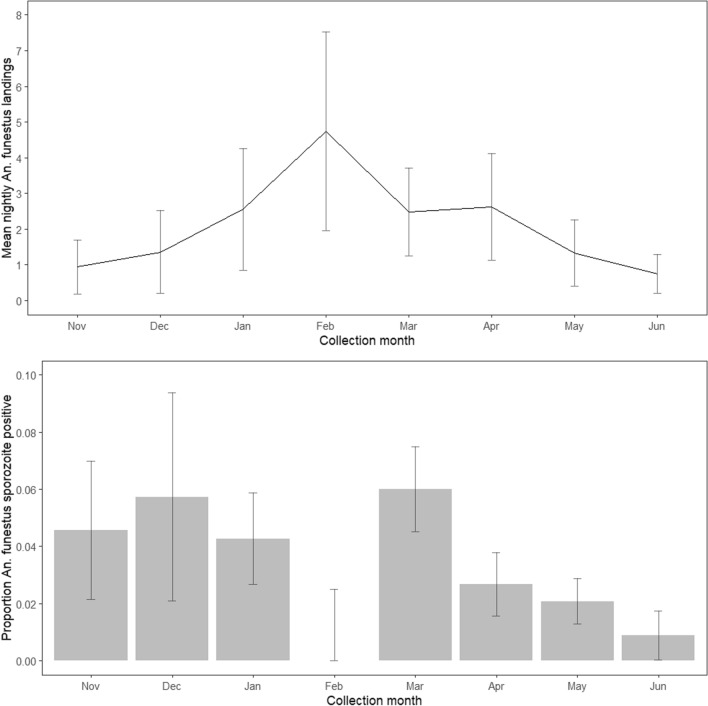
Line chart describing the mean nightly *An*. *funestus* landing on human landing collectors by month (top), and bar chart displaying proportion of tested *An*. *funestus* found sporozoite-positive by month (bottom)

ELISA testing was completed on 4768 female *An*. *funestus* (1800 from HLC, 2968 from CDC light trap collections) to identify presence of *P*. *falciparum* circumsporozoite (CS) protein. Pooling results for both trapping methods over all collection nights and clusters, 159 (3.3%) were CS-positive and subsequently referred to as sporozoite-positive (3.5% among HLC samples, 3.2% among CDC light trap samples). Sporozoite positivity and mean landing rates by month for all clusters pooled are shown in Fig. 2. Two clusters had no sporozoite positive samples identified. The highest sporozoite prevalence was 11.4% (9/79) in cluster 80 (Table 2).

**Table 2:** Cluster-level summary of *An*. *funestus* *P*. *falciparum* sporozoite positive samples (collected by HLC or CDC light trap), mean female *An*. *funestus* landing rate by night by collector for indoor and outdoor locations and directly-measured entomological inoculation rate calculated from overall means of indoor and outdoor *An*. *funestus* landing rates per night

Cluster	*An*. *funestus* tested by ELISA	Sporozoite positive (%)	*N* trap nights	Mean nightly indoor *An*. *funestus* landings	Mean nightly outdoor *An*. *funestus* landings	Mean nightly *An*. *funestus* landings	Mean nightly directly-measured EIR
23	232	13 (5.60)	153	4.39	4.62	5.05	0.252
10	630	18 (2.86)	150	10.18	5.74	7.45	0.228
56	222	11 (4.95)	143	3.77	2.50	2.79	0.155
80	79	9 (11.39)	154	1.05	0.97	0.89	0.115
38	501	11 (2.20)	159	5.26	4.53	5.16	0.107
71	352	15 (4.26)	128	2.37	2.30	2.24	0.100
27	232	10 (4.31)	150	2.34	1.78	2.37	0.089
51	399	8 (2.01)	136	5.05	2.04	3.56	0.071
40	190	13 (6.84)	116	1.18	0.77	1.03	0.067
69	181	10 (5.52)	156	1.65	0.73	1.18	0.066
67	132	5 (3.79)	158	1.41	0.98	1.21	0.045
19	233	10 (4.29)	152	1.26	0.76	0.93	0.043
59	613	8 (1.31)	163	2.63	2.62	2.58	0.034
74	309	4 (1.29)	158	2.29	1.68	1.91	0.014
26	38	3 (7.89)	158	0.08	0.36	0.28	0.017
20	186	7 (3.76)	150	0.40	0.25	0.33	0.012
79	123	2 (1.63)	150	0.72	0.16	0.39	0.007
29	74	2 (2.70)	135	0.18	0.10	0.14	0.004
4	14	0 (0.00)	149	0.16	0.13	0.16	0.000
7	28	0 (0.00)	150	0.13	0.07	0.10	0.000
Overall mean	4768	159 (3.33)	2968	2.32	1.66	1.99	0.072

The cluster-specific directly-measured EIR ranged from zero to 0.25 infectious *An*. *funestus* bites per person per night (ib/p/n) during the malaria transmission season. The overall mean directly-measured EIR for individuals exposed to biting from 18:00 to 06:00 at the study site was 0.07 ib/p/n during the malaria season. Note that these EIR estimates average indoor and outdoor *An*. *funestus* landing rates, and do not consider an individual’s usual behaviour or use of personal protection against mosquito bites.

**Fig. 3:**
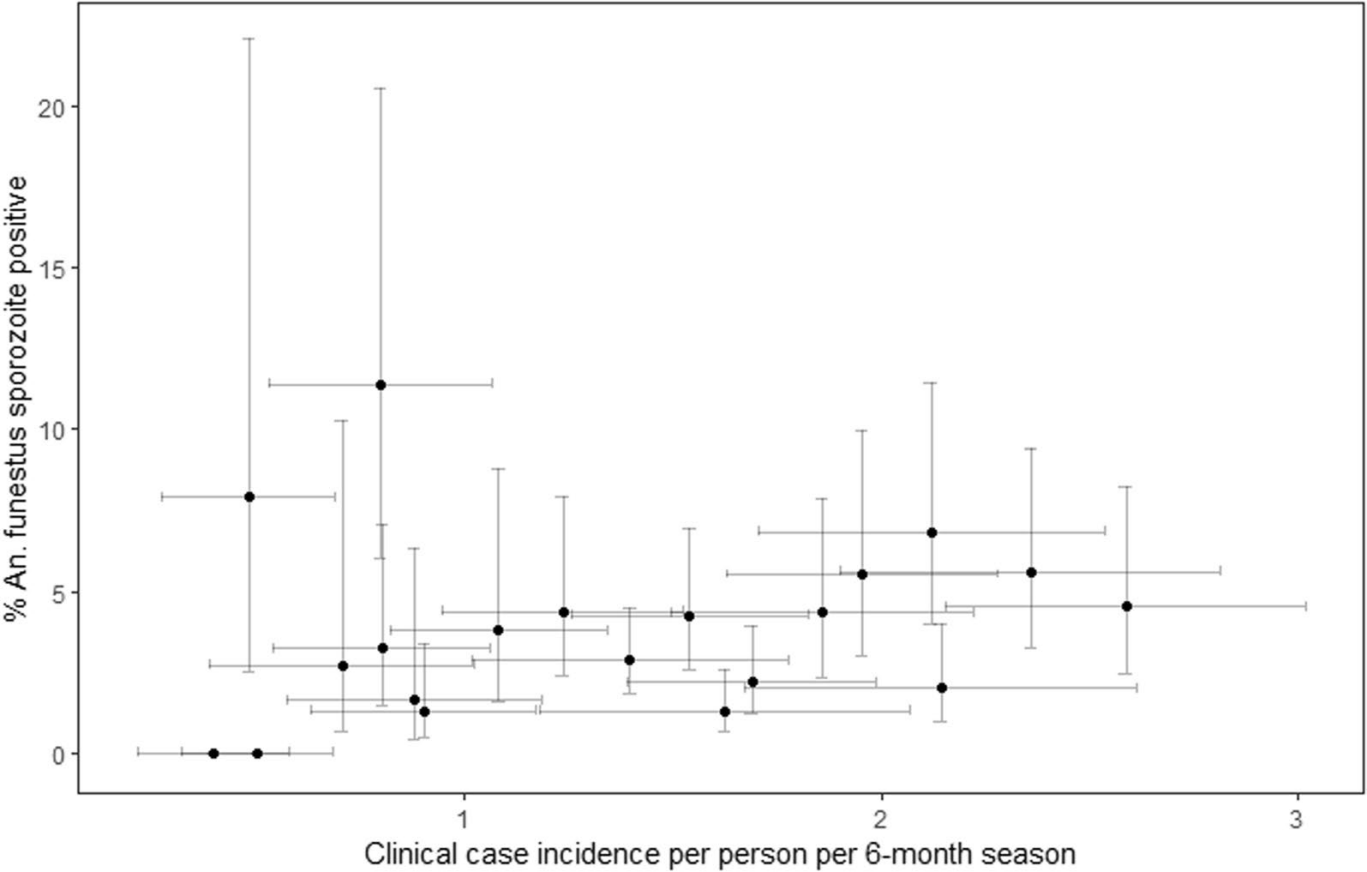
Scatter plot of cluster-level sporozoite prevalence among *An*. *funestus* collected by human landing catch and CDC light trap versus mean incidence of clinical malaria per child per 6 month transmission season by cluster. Grey lines indicate 95% confidence interval

Cluster-level summaries of sporozoite prevalence show a weak positive correlation (Pearson *r* = 0.169) with clinical malaria incidence by cluster, as estimated from the two seasonal cohorts of children aged 1–14 years (Fig. 3).

### Human behaviour-adjusted *An*. *funestus* biting rates and EIR

Linking reported locations during the period 18:00 to 06:00 from 4857 total follow-up cohort visits to 1237 children with the relevant hourly *An*. *funestus* biting rate for the cluster and month estimated that a total of 2488 *An*. *funestus* bites would have been experienced by this population on the night before the follow-up visit.

There was large variation in estimated human behaviour-adjusted *An*. *funestus* bites received per child between the study clusters. Human behaviour-adjusted nightly biting ranged by cluster from mean 1.65 b/p/n [95% confidence interval (95% CI 0.16–3.15)] in cluster 59 to 0.18 b/p/n in cluster 26 (95% CI 0.00–0.25). Human behaviour-adjusted nightly EIR ranged from 0 to 0.05 ib/p/n by cluster, equivalent to 0 to 12.34 total infectious bites per person over a 6 month transmission season (Table 3). Mean human behaviour-adjusted EIR across the whole study site was 0.02 ib/p/n.

**Table 3:** Estimated *An*. *funestus* infectious bites per 6 month transmission season (January–June) by cluster experienced by human landing collectors (‘directly measured’), and estimated to have been experienced by children (‘human behaviour-adjusted’). Human behaviour adjusted entomological inoculation rate (EIR) per 6 month transmission season is further stratified among children using ITNs and those not using ITNs

Cluster	Mean ‘directly measured’ EIR per person per 6 month season	% child-visits where child slept under ITN	Mean ‘human behaviour-adjusted’ EIR per person per 6 month season	Mean ‘human behaviour-adjusted’ EIR per person per 6 month season among children using ITN	Mean ‘human behaviour-adjusted’ EIR per person per 6 month season among children not using ITN
56	41.05	94.4	8.07	5.36	58.34
23	38.83	78.7	12.34	2.48	39.94
51	17.84	93.0	2.95	0.61	33.68
80	30.94	88.4	4.31	1.54	31.14
38	26.81	87.2	3.43	1.71	25.44
69	14.93	72.3	8.22	1.67	24.91
71	20.14	82.4	6.73	3.40	23.02
27	13.76	78.7	5.37	1.00	20.77
10	47.99	95.0	5.19	3.47	19.27
40	9.31	87.1	2.08	0.41	12.17
67	11.22	67.5	5.28	1.90	11.33
59	6.82	70.1	3.20	1.88	6.47
74	5.77	88.1	0.89	0.28	5.76
19	8.72	89.8	0.44	0.17	4.36
79	2.21	87.5	0.54	0.04	3.23
20	1.55	90.8	0.21	0.05	2.32
26	3.22	85.1	0.37	0.02	1.70
29	0.70	58.9	0.37	0.01	0.78
4	0.00	93.6	0.00	0.00	0.00
7	0.00	77.6	0.00	0.00	0.00
Overall mean	15.09	83.2	3.52	1.34	13.59

Children using ITNs received on average 0.21 *An*. *funestus* bites per night (95% CI 0.00–0.80), with 84% of those bites occurring while they were outside in the evening or early morning. Across a 6 month transmission season from January to June, it was estimated that children using ITNs consistently received a total of 37.4 *An*. *funestus* bites, 1.3 of which were estimated to be *P*. *falciparum* sporozoite-positive (Table 4). Children who did not use ITNs experienced far higher biting rates, receiving an estimated 2.0 *An*. *funestus* bites per night (95% CI 0.42, 3.60), only 9% of which occurred outside. Across a 6 month transmission season, children not sleeping under ITNs received 356 *An*. *funestus* bites if they consistently did not use an ITN, 13.6 of which were estimated to be sporozoite-positive.

**Table 4:** Estimated *An*. *funestus* bites and infectious bites experienced over a 6 month transmission season among children who sleep under an ITN and those who do not use an ITN

	Children sleeping under ITN on the previous night	Children not using ITN on the previous night
Number child-visits	4040	817
Estimated *An*. *funestus* bites		
Mean *An*. *funestus* bites per person per night over the period January–June (95% CI)	0.21 (0.00–0.80)	2.01 (0.42–3.60)
Total *An*. *funestus* bites per person per 6-month season (95% CI)	37.4 (0–81.5)	355.7 (238.8–472.6)
% of *An*. *funestus* bites occurring outdoors	83.81%	9.11%
Estimated sporozoite-positive *An*. *funestus* bites		
Mean *An*. *funestus* infectious bites (entomological inoculation rate, EIR) per person per night over the period January–June (95% CI)	0.007 (0.00–0.03)	0.08 (0.01–0.16)
Estimated total infectious *An*. *funestus* bites (EIR) per person per 6-month season (95% CI)	1.34 (0–3.09)	13.59 (7.43–19.74)
% of infectious *An*. *funestus* bites occurring outdoors	85.63%	7.98%

Mean bites are calculated according to reported location (outdoors or indoors) and hourly cluster-, location- and month-specific *An*. *funestus* landing rates among child-observations who reported using an ITN on the previous night (*N* = 4040), and those not using an ITN on the previous night (*N*=817). Estimated infectious bites use a cluster-level sporozoite positivity rates that are static over time

**Fig. 5:**
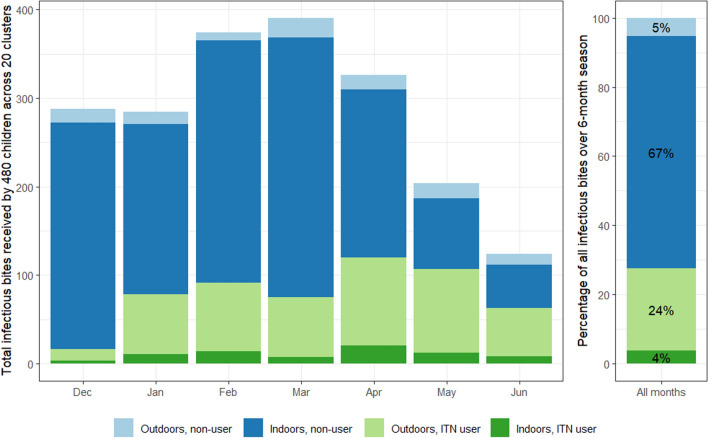
Stacked bar charts representing the total infectious *An*. *funestus* bites (left panel) received each month by the whole study population across 20 clusters, and (right panel) the aggregate infectious *An*. *funestus* bites over the full study period. Cluster study population was standardized to 24 children, reported cluster-month ITN use applied to determine final number of ITN users and non-users within each cluster, with each child receiving the mean nightly indoor and outdoor bites for the specific month according to their ITN use status. Total bites were aggregated for each calendar month and across all 20 clusters.

The highest number of infectious bites estimated to be received over a 6 month transmission season by a child not using an ITN was 58, in cluster 56 (Fig. 4, Table 3). The highest estimated number of infectious bites experienced by a child using an ITN was 5.4 total over a 6 month season (in cluster 56): this was a higher EIR than that experienced by children not using ITNs in seven clusters. Descriptions of human behaviour-adjusted total bites and EIR by night and 6 month season are further stratified by cluster IRS status and trial arm in Tables S1 and S2, respectively. When aggregating all infectious bites received by the study population according to cluster biting rates and reported ITN usage, the largest number of infectious bites are received indoors by children not using ITNs, with the second largest number of infectious bites received by ITN users while they are outside (Fig. 5, Fig. S1).

**﻿Fig. 4:**
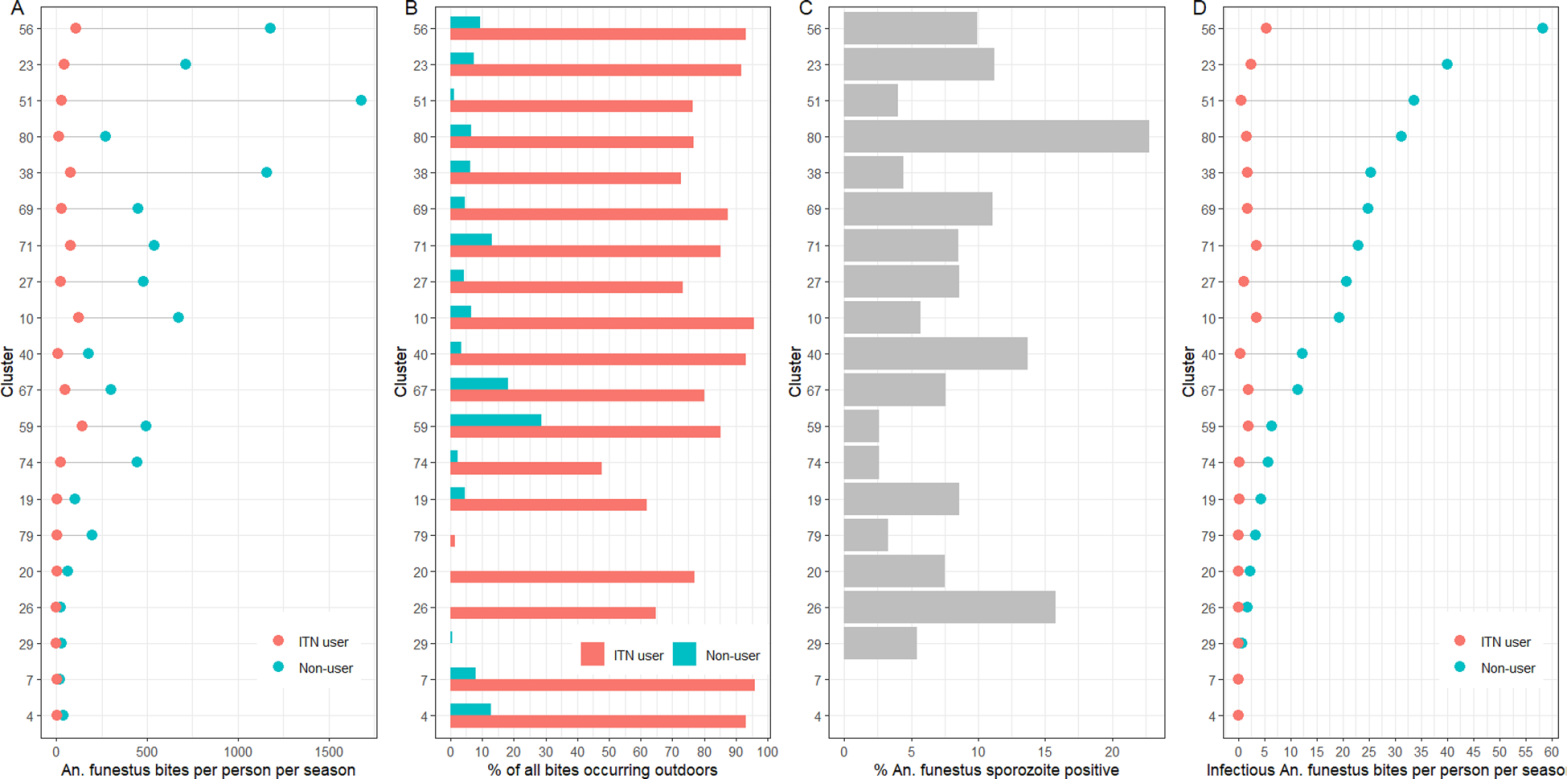
Summary of key indicators by cluster. **A** Dot plot describing estimated behaviour-adjusted *An*. *funestus* bites per person per 6 month transmission season (January-June) received by ITN users and non-users in each cluster. **B** Bar chart describing the proportion of all behaviour-adjusted *An*. *funestus* bites that ITN users and non-ITN users are estimated to have received while outside. **C** Proportion of all *An*. *funestus* by cluster that were positive for *P*. *falciparum* circumsporozoite protein when tested by ELISA. **D** Dot plot describing estimated behaviour-adjusted infectious *An*. *funestus* bites per person (entomological inoculation rate) per 6-month transmission season (January-June) received by ITN users and non-users in each cluster.

### Proportion of bites that are preventable using existing tools

During the 6 month malaria transmission season, children consistently using ITNs received on average 1.34 (95% CI 0.00–3.09) total infectious bites, while those children not consistently using ITNs were estimated to receive 13.59 (95% CI 7.43–19.74) total infectious bites over the 6 month period. Approximately 89.6% (95% CI 58.8–100) of infectious *An*. *funestus* bites in this study site among children were estimated to be prevented by ideal use of ITNs. Among the infectious bites that cannot be prevented by use of ITNs, most bites occur while children are outside (85.6%). While the overall majority of bites in this setting could potentially be prevented through the use of ITNs, persistent transmission still comprises an estimated 1.3 infectious *An*. *funestus* bites per person over a 6 month transmission season.

## Discussion

This secondary analysis of entomological surveillance data collected during the ATSB trial in Western Province Zambia indicates that in children 90% of infectious *An*. *funestus* bites can be prevented by perfect use of ITNs, since biting pressure remains highest indoors and during usual sleeping hours. Children who do not sleep under ITNs receive the majority of bites while indoors (91%) and are estimated to receive 13.6 infectious *An*. *funestus* bites over a 6 month transmission season. Among children using ITNs, the number of bites received is much lower, but bites are primarily experienced while outside (84%), and children consistently using ITNs are estimated to have received on average 1.3 infectious *An*. *funestus* bites over a 6 month season. Consequently, while the protective effect of ITNs is high in this setting among children, persistent malaria transmission largely occurs outdoors and represents a substantial risk to the population and public health burden, considering the high level of *An*. *funestus* biting and the high sporozoite positivity of the vector.

Directly-measured EIR, generated directly from the mean of indoor and outdoor landing rates reported by HLC collectors and sporozoite-positivity, was estimated at 0.07 infectious bites per person per night across the study site. However, use of human behaviour-adjusted EIR, which combines sporozoite-positivity, each child’s reported location (outside, inside but not under ITN, or inside and under an ITN) by hour and cluster- and month-specific hourly biting rates to estimate the actual bites that would be received by an individual, reveals further nuances. Among children who do not use ITNs, mean human-adjusted EIR was estimated at 0.08 infectious bites per person per night, slightly higher than the directly-measured EIR (0.07), as a result of children being exposed to the relatively higher indoor biting rates throughout the night. However human-adjusted EIR among ITN users is much lower, at 0.007 infectious bites/person/night, reflecting the personal protection offered by ITNs while indoors and sleeping under an ITN.

Substantial heterogeneity was observed in landing rates and sporozoite positivity between clusters across the study site, such that ITN users in the highest biting rate clusters were estimated to receive more infectious bites per night than children not using ITNs in relatively lower biting rate clusters. This emphasises that even in a setting with relatively high overall ITN use (83% among participating children, and >70% among the general population), children may still experience substantial exposure to biting before entering their ITN for the night and that even in high transmission settings there remains heterogeneity in malaria risk over time and space.

Persistent, or residual, transmission in this setting is estimated as a human behaviour-adjusted EIR of 1.3 infectious bites per person per 6 month season among ITN users, despite very high ITN household possession (>90%) and use (>70%). Malaria prevalence has been shown to have an approximately linear relationship with the log of annual EIR [25], highlighting that EIR needs to be reduced well below 1 per year to have any observable impact on malaria prevalence. At our study site, malaria prevalence was 51.9% among individuals aged 6 months and older in the baseline year [7] (prior to top-up ITN distributions and assignment of ATSB to the intervention arm) and remained just over 50% throughout the trial [5]. Corresponding to this high infection prevalence in the human population, 3.3% of all tested *An*. *funestus* were CS-positive. While ITN use is high in this setting, it is clear that persistent malaria transmission contributes to maintaining a high parasite load and public health burden in the population.

Our estimate that 9% of all bites received by individuals who do not use an ITN occur outside is consistent with the findings of a meta-analysis describing the role of outdoor biting [11]. However, among individuals using ITNs, outdoor biting contributes most of the exposure to biting since our study population, consisting only of children, usually go under their ITNs immediately after entering their sleeping structure. Our behavioural data describing ITN use and locations during the evening is limited to children, and it is likely that adults may go to bed later and spend additional time outside in the evenings, extending the period when they are exposed to persistent infectious biting, and playing a key role in sustaining very high malaria transmission across this population. The proportion of bites that can be prevented by using an ITN is likely to be substantially lower for adults than the estimate of 90% in children, whose use of ITNs is more effective because they likely spend more time under them than adults during the night. Furthermore, our behavioural data is collected by interview and may not correlate exactly with behavioural data collected by direct observation.

Our analysis method combines indicator estimates generated at different levels, and while we have attempted to handle uncertainty in landing rates, sporozoite positivity and resulting EIR using delta-method approximations, the uncertainties presented should be interpreted cautiously owing to challenges in estimating and propagating covariance structures through the various levels of analysis. Considering the growing interest in estimating human behaviour-adjusted EIR [26–33], which joins estimates of biting rates with estimates of behaviour, often from slightly different individuals or populations, there is a need for further methodological guidance describing best practices for uncertainty generation in these calculations.

The current analysis focusses on personal protection provided by sleeping under ITNs, and community protection effects have not been explicitly estimated. However, since ITN ownership at the study site was >90% and ITN use >70%, the mosquito landing rates captured at sampling locations inherently reflect any impact of ITN on overall vector abundance. *An*. *funestus* at the study site has high levels of pyrethroid resistance, but resistance has not been detected against the chemical used for IRS in this location, clothianidin, or other IRS chemicals, such as pirimiphos-methyl [7]. There was little difference in estimating biting rates when stratifying clusters according to whether they were targeted for IRS, suggesting a limited effectiveness of IRS at the study site that may be attributable to challenges in achieving high coverage in a rural setting with dispersed settlements, optimal timing of IRS campaigns, or other factors. ITNs used at the trial site were a mixture of deltamethrin (Permanet 2.0) and alpha cypermethrin plus piperonyl butoxide (PBO, Veeralin) types [7]. It is possible that the ITNs used had insufficient insecticidal effectiveness to reduce the vector population, resulting in the relatively high observed proportion of *An*. *funestus* that were sporozoite positive, and high EIR experienced by non-ITN users as a result of limited mass effect of ITNs. However, modelling studies indicate that even in settings with high pyrethroid resistance, there are still likely to be community-protection effects from high levels of pyrethroid ITN use [34]. Further studies are required to assess if the extent of persistent malaria transmission in this site could be reduced by use of bioefficacious ITNs or IRS, or combinations of these interventions.

Our estimates of directly-measured and human behaviour-adjusted EIR were limited by conducting mosquito trapping between 18:00 and 06:00 only. Hourly biting rates in the period 05:00–06:00 had not yet dropped to zero, suggesting that our data underestimated biting occurring in the early morning. Recent 24 h mosquito trapping activities at the study site indicate that biting does occur beyond the usual sampling period of 18:00–06:00, and that expanding the trapping period beyond this period increases measured nightly biting rates by ~5% (B. Chanda, personal communication). Further research is needed to clarify how vector biting in the late morning, early evening, and at locations away from households might contribute to persistent malaria transmission in Western Zambia.

Nightly biting rates and sporozoite positivity in our study are broadly similar to those reported from other locations where *An*. *funestus* is the primary vector [17, 18, 35, 36], but much lower than biting rates experienced in west African settings where *An*. *coluzzii* is the primary vector [33]. In many settings in southern and eastern Africa, *An*. *funestus* has become the dominant malaria vector, replacing *An*. *gambiae* s.l. [37, 38]. Furthermore, the decreases in sporozoite positivity and corresponding EIR by *An*. *gambiae* s.l. since 2000 have not been observed in *An*. *funestus* [37]. Recent evidence of indoor morning *An*. *funestus* biting after people rise for the day [15, 31], and during school hours [16], further emphasise the challenge of addressing persistent transmission in settings where *An*. *funestus* is the primary vector*.*

## Conclusions

In this high transmission setting in western Zambia the majority of *An*. *funestus* bites are in theory preventable by use of ITNs, but it is not programmatically feasible to achieve 100% ITN coverage among the population. For the minority of individuals who do not use an ITN in the current context and receive the majority of all infectious *An*. *funestus* bites, there remains a need for alternative indoor interventions to protect this population from very high biting rates, as well as tools that effectively control *An*. *funestus* populations and provide community-level protection. In addition, the remaining persistent, or residual, malaria transmission that occurs beyond the hours when individuals are sleeping under ITNs still comprises over one infectious *An*. *funestus* bite per person per 6 month transmission season and is likely to contribute to persistent high malaria burden in similar settings. New and more effective vector control tools that reduce *An*. *funestus* inoculation rates are required to continue the progress against malaria that was seen prior to 2015. Further field studies are also needed to better understand how very high malaria transmission can be sustained even with very high coverage of existing vector control tools.

## Supplementary Information

Supplementary Material 1.

## Data Availability

De-identified data from the current study are available from the corresponding author on reasonable request.
