# Supplementary material for: Why does malaria transmission continue at high levels despite universal vector control? Quantifying persistent malaria transmission by Anopheles funestus in Western Province, Zambia
Source: Parasit Vectors. 2024 Oct 14;17:429. doi: 10.1186/s13071-024-06457-5 (PMC11476814; doi:10.1186/s13071-024-06457-5)

**Table S1**: Summary of biting rates experienced by net users & non users, stratified by clusters targeted for IRS, and those not targeted for IRS

|  | **IRS targeted clusters** | | |  | **Clusters not targeted for IRS** | | |
| --- | --- | --- | --- | --- | --- | --- | --- |
|  | **All children** | **ITN users** | **Non-users** |  | **All children** | **ITN users** | **Non-users** |
| Number child-visits | 2055 | 1501 | 554 |  | 2802 | 2539 | 263 |
| ***Estimated An. funestus bites*** |  |  |  |  |  |  |  |
| Mean *An. funestus* bites per person per night (95% CI) | 0.72  (0.00-1.56) | 0.28  (0.00-0.87) | 1.91  (0.40-3.42) |  | 0.36  (0.00-1.07) | 0.17  (0.00-0.77) | 2.21  (0.46-3.96) |
| Total bites per person per season (January to June) (95% CI) | 129.77  (66.33-193.22) | 42.03  (0.0-85.25) | 331.07  (220.74-441.40) |  | 67.59  (15.08-120.11) | 31.65  (0.0-75.93) | 414.40  (280.38-548.42) |
| % of *An. funestus* bites occurring outdoors | 28.65% | 85.65% | 12.00% |  | 37.47% | 82.82% | 4.09% |
| ***Estimated sporozoite-positive An. funestus bites*** |  |  |  |  |  |  |  |
| Mean *An. funestus* infectious bites per person per night (95% CI) | 0.03  (0.00-0.07) | 0.009  (0.00-0.03) | 0.08  (0.00-0.15) |  | 0.01  (0.00-0.04) | 0.006  (0.00-0.03) | 0.08  (0.00-0.17) |
| Estimated infectious *An. funestus* bites per person per season (January to June) (95% CI) | 5.20  (2.20-8.20) | 1.46  (0.0-3.24) | 13.37  (7.66-19.08) |  | 2.40  (0.17-4.63) | 1.16  (0.0-2.89) | 14.43  (7.07-21.80) |
| % of infectious *An. funestus* bites occurring outdoors | 24.20% | 85.96% | 9.17% |  | 40.57% | 85.66% | 5.61% |

**Table S2:** Summary of biting rates experienced by net users & non users, stratified by ATSB trial arm

|  | **ATSB arm clusters** | | |  | **Control arm** | | |
| --- | --- | --- | --- | --- | --- | --- | --- |
|  | **All children** | **ITN users** | **Non-users** |  | **All children** | **ITN users** | **Non-users** |
| Number child-visits | 2446 | 1998 | 448 |  | 2411 | 2042 | 369 |
| ***Estimated An. funestus bites*** |  |  |  |  |  |  |  |
| Mean *An. funestus* bites per person per night (95% CI) | 0.46  (0.00-1.16) | 0.23  (0.00-0.81) | 1.48  (0.23-2.72) |  | 0.57  (0.00-1.39) | 0.19  (0.00-0.80) | 2.66  (0.65-4.66) |
| Total bites per person per season (January to June) (95% CI) | 88.23  (35.45-141.02) | 43.14  (0.00-86.35) | 272.06  (178.80-365.32) |  | 99.28  (38.30-160.27) | 31.91  (0.00-76.95) | 457.31  (311.24-603.39) |
| % of *An. funestus* bites occurring outdoors | 41.61% | 90.03% | 10.10% |  | 26.37% | 75.69% | 8.46% |
| ***Estimated sporozoite-positive An. funestus bites*** |  |  |  |  |  |  |  |
| Mean *An. funestus* infectious bites per person per night (95% CI) | 0.02  (0.00-0.06) | 0.01  (0.00-0.04) | 0.07  (0.00-0.15) |  | 0.02  (0.00-0.04) | 0.005  (0.00-0.02) | 0.08  (0.00-0.17) |
| Estimated infectious *An. funestus* bites per person per season (January to June) (95% CI) | 4.13  (1.18-7.08) | 1.92  (0.00-4.15) | 13.15  (7.18-19.13) |  | 2.89  (0.79-4.98) | 0.77  (0.00-2.06) | 14.04  (7.63-20.44) |
| % of infectious *An. funestus* bites occurring outdoors | 39.44% | 89.02% | 9.86% |  | 21.70% | 77.45% | 5.80% |

**Table S3:** Summary characteristics of children included in biting rate analysis, described according to their reported ITN use over all visits: consistent ITN users, inconsistent ITN users, and those who reported not using and ITN the previous night over all their visits

| **Characteristic** | **Consistent ITN users (N=743)** | **Inconsistent ITN users (N=413)** | **Non-users of ITNs (N=93)** | **p-value^1^** |
| --- | --- | --- | --- | --- |
| Male (%) | 342 (46.0) | 192 (46.5) | 50 (53.8) | 0.367 |
| Age:  <5 years  5-9 years  10-14 years | 315 (42.4)  299 (40.2)  129 (17.4) | 103 (24.9)  155 (37.5)  155 (37.5) | 10 (10.8)  39 (41.9)  44 (47.3) | <0.001 |
| IRS reported received at household^2^ | 189 (25.5) | 135 (37.8) | 39 (41.9) | 0.003 |
| NMEC microplan assigned to receive IRS | 282 (38.0) | 231 (55.9) | 77 (82.8) | <0.001 |
| Child sleeps in ‘closed’ structure (closed eaves and all windows & doors have screens or shutters) | 112 (15.1) | 66 (16.0) | 17 (18.3) | 0.702 |
| Household has at least 1 ITN for every 2 people | 316 (42.3) | 115 (27.8) | 11 (13.4) | <0.001 |
| Household wealth  Poorest  2^nd^ poorest  Median  2^nd^ richest  Richest | 97 (13.1)  173 (23.3)  111 (14.9)  192 (25.8)  170 (22.9) | 57 (13.8)  98 (23.7)  66 (16.0)  100 (24.2)  92 (22.3) | 28 (30.1)  17 (18.3)  18 (19.4)  14 (15.1)  16 (17.2) | 0.002 |

^1^From Chi-squared test, or Fishers’ exact test

^2^IRS status unknown for 4 participants: 3 who always used ITN, 1 who inconsistently used ITN;

**Figure S1** Stacked bar charts representing the total infectious *An. funestus* bites received each month by the whole study population across 20 clusters. Cluster study population was standardized to 24 children, reported cluster-month ITN use applied to determine final number of ITN users and non-users within each cluster, with each child receiving the mean nightly indoor and outdoor bites for the specific month according to their net use status. Total bites were aggregated for each calendar month within each cluster.


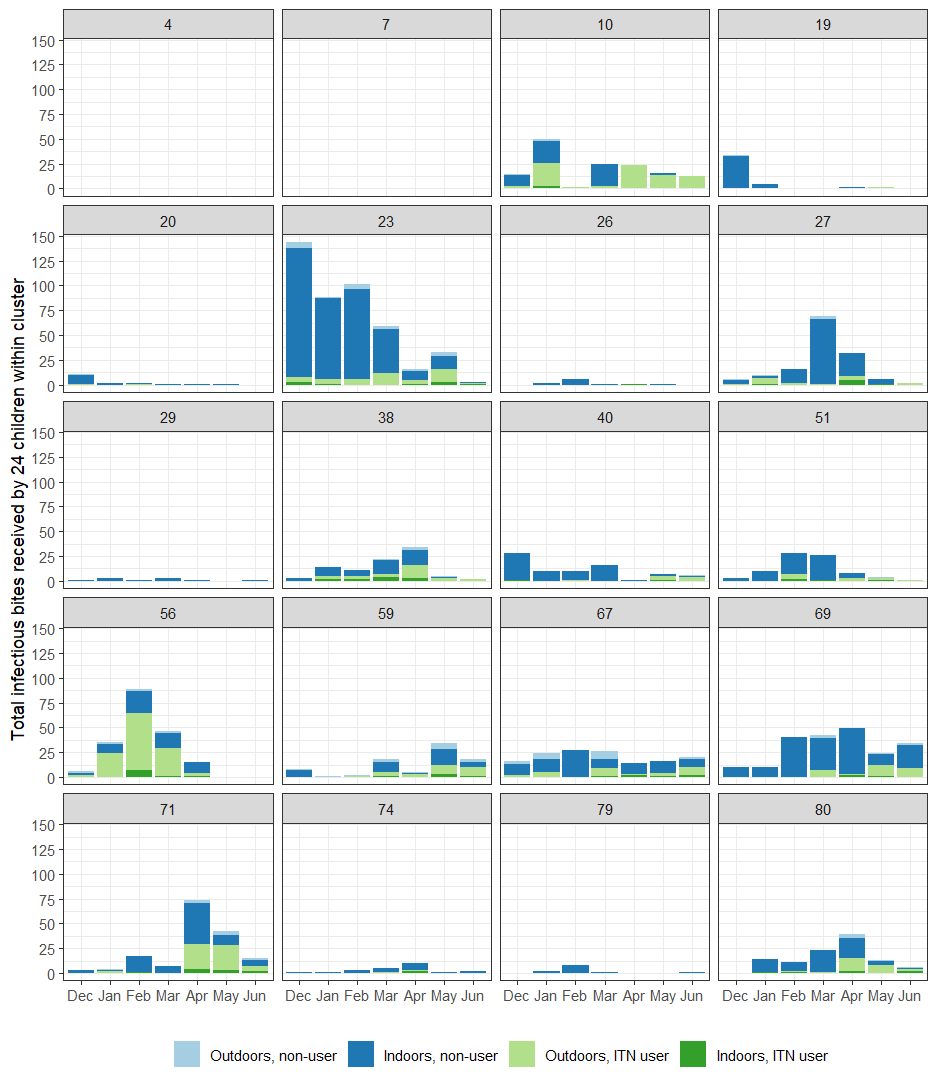

Supplement: Supplementary file 1 — Supplementary Material 1. [file 13071_2024_6457_MOESM1_ESM.docx]
